# Patient-Specific Pluripotent Stem Cells in Neurological Diseases

**DOI:** 10.4061/2011/212487

**Published:** 2011-07-03

**Authors:** Serpen Durnaoglu, Sermin Genc, Kursad Genc

**Affiliations:** Department of Neuroscience, Health Science Institute, Dokuz Eylül University, Inciralti, 35340 Izmir, Turkey

## Abstract

Many human neurological diseases are not currently curable and result in devastating neurologic sequelae. The increasing availability of induced pluripotent stem cells (iPSCs) derived from adult human somatic cells provides new prospects for cellreplacement strategies and disease-related basic research in a broad spectrum of human neurologic diseases. Patient-specific iPSC-based modeling of neurogenetic and neurodegenerative diseases is an emerging efficient tool for *in vitro* modeling to understand disease and to screen for genes and drugs that modify the disease process. With the exponential increase in iPSC research in recent years, human iPSCs have been successfully derived with different technologies and from various cell types. Although there remain a great deal to learn about patient-specific iPSC safety, the reprogramming mechanisms, better ways to direct a specific reprogramming, ideal cell source for cellular grafts, and the mechanisms by which transplanted stem cells lead to an enhanced functional recovery and structural reorganization, the discovery of the therapeutic potential of iPSCs offers new opportunities for the treatment of incurable neurologic diseases. However, iPSC-based therapeutic strategies need to be thoroughly evaluated in preclinical animal models of neurological diseases before they can be applied in a clinical setting.

## 1. Introduction

Human neurological diseases including stroke, neurodegenerative disorders, neurotrauma, multiple sclerosis (MS), and neurodevelopmental disorders are caused by a loss of neurons and glial cells in the brain or spinal cord. They usually cause morbidity and mortality as well as increase social and economic burdens of patients and their caregivers [1]. Stroke is one of the leading cause, of death and the primary cause of morbidity and long-term neurological disability. The burden of the age-related neurodegenerative diseases including Alzheimer's disease (AD), other dementias, and Parkinson's disease (PD) is expected to increase dramatically as the life expectancy and aging population rise worldwide [2]. Neurodegenerative diseases represent a large group of heterogeneous disorders characterized by progressive degenerative loss of specific neuron subtypes over time: cortical neurons in AD, dementia with Lewy bodies, or frontotemporal lobar degeneration, midbrain dopaminergic neurons in PD, striatal GABAergic neurons and cortical neurons in Huntington's disease (HD), cerebellar neurons in spinocerebellar degeneration, and upper and lower motor neurons in amyotrophic lateral sclerosis (ALS) [3]. Nonneuronal cells also contribute to the progression of neurodegeneration [4]. By contrast, rapid cell loss and destruction of larger areas of central nervous system (CNS) tissue are seen in acute lesions, such as in acute ischemic or hemorrhagic stroke, traumatic brain injury, and spinal cord injury (SCI). The CNS has limited capacity of regenerating lost tissue in both cases and require strategies to manage the neurological deficits caused by the neural tissue destruction. However, conventional therapies of many neurological diseases provide only limited benefit by alleviating certain symptoms. The chronic use of these drugs is often associated with serious side effects, and none seems to modify the natural course of these diseases [5]. Many attempts have been made to develop neuroprotective drugs to reduce the CNS injury, but the translation of neuroprotection from experimental therapies to clinical setting has not been very successful [6]. Although the adult brain contains small numbers of stem cells in restricted areas and acute neurological insults stimulate a basal rate of neural progenitor/precursor proliferation and differentiation, they do not contribute significantly to functional recovery. Moreover, adult neurogenesis may be defective in neurodegenerative diseases [7]. Along with the development of stem cell technologies, transplantation of stem cells or their derivatives is a future therapeutic option for human neurological diseases. 

Stem cells are characterized by the ability to renew themselves (self-renewal) through mitotic cell division and differentiate into a diverse range of specialized cell types [8]. These cells are classified into three types according to their capacity to differentiate into specialized cells (potency). The first type is totipotent stem cells, which can be implanted in the uterus of a living animal and give rise to an entire, viable organism. The second type is pluripotent stem cells such as embryonic stem cells (ESCs) that are isolated from the inner cell mass of blastocysts and induced pluripotent stem cells (iPSCs) artificially derived from a nonpluripotent cell, typically an adult somatic cell through reprogramming. 

Pluripotent stem cells can give rise to every cell of an organism except extraembryonic tissues, such as placenta [8]. ESCs can become all cell types while adult stem cells (ASCs) are thought to be limited in differentiating into different cell types of their tissue of origin. ASCs are found rarely in mature tissues; therefore, isolation of these cells from adult tissue is challenging; however, ESCs can be grown in cell culture. This difference is crucial for stem cell replacement therapies because large numbers of cells are needed for therapeutic applications. The tissues derived from the patient's own ASCs are currently believed less likely to initiate rejection after transplantation. This is significant for solving immune rejection problem of cell replacement therapies.

The third type is multipotent stem cells that only generate specific lineages of cells. Neural stem cells (NSCs) are multipotent stem cells which are derived from neural tissues [9]. These cells are self-renewing and differentiate into lineage-specific neural precursor or progenitor cells (NPCs) that can give rise to all cell types (neurons, astrocytes, and oligodendrocyes) of the nervous system through asymmetric cell division. 

The potential applications of stem cell therapies for treating neurological disorders are enormous. Many laboratories are focusing on stem cell treatments for CNS diseases, including SCI, stroke, ALS, PD, MS, and epilepsy [10–16]. 

 Finally, clinical trial on stem cell therapy for treatment of neurological disorders was started. Autologous bone marrow stem cells and mesenchymal stem cells are used for treatment of amyotrophic lateral sclerosis. In addition, Geron Corp has started clinical trial using hESC-derived oligodendrocyte progenitor cells for spinal cord injury (http://clinicaltrials.gov/). However, ethical concerns, immune rejection of grafted stem cells, and tumor formation limit the use of human ESCs. 

The development of iPSCs in recent years may bypass the ethical controversies and rejection problem using autologous stem cells, albeit tumor formation stands as a challenge for cell-replacement therapy [17]. Various neural cell types have been differentiated from human or rodent iPSCs generated by the reprogramming of different somatic cells, mainly skin fibroblasts [18–20]. IPSCs have also been differentiated to NPCs [21, 22]. In terms of cell morphology and pluripotency, iPSCs closely resemble ESCs. Several groups have successfully generated a wide range of iPSCs from patients with neurodevelopmental and neurodegenerative diseases [23–25]. Patient-specific iPSCs overcome the graft rejection problem providing an autologous cell source. Genetic correction of patient-specific iPSCs derived from the patients with neurogenetic disorders may be required before the transplantation process. Patient-specific iPSCs also represent a valuable tool to dissect the poorly understood mechanisms of neurogenetic and neurodegenerative diseases. As mentioned above, animal studies alone cannot unravel the complexities of the human brain and alternative approaches for disease modeling are required. The failure to translate the promising results of preclinical neuroprotection studies to the clinic setting may be due to many factors including species differences, brain complexity, age, patient variability, and disease-specific phenotypes that cannot easily be modelled in chosen nonhuman experimental systems [26, 27]. Cellular modeling studies and chimeric mouse models based on iPSCs may overcome these barriers [12, 28]. Finally, patient-specific iPSCs may be most relevant cell source for drug screening and development as they take into consideration the patient's background, the affected cell type, and the developmental time [24, 29]. In this review, we summarized the recent advancements in iPSC generation, their capacity for differentiation toward neural lineages, and iPSC-based transplantation and disease modeling attempts for neurological diseases.

## 2. The Generation of iPSCs

### 2.1. History of iPSCs

IPSCs were initially derived from mouse embryonic and adult fibroblasts by overexpression of particular transcription factors, which have become famous as the “Yamanaka factors.” To identify transcriptional regulators capable of reprogramming adult somatic cells into pluripotent cells, Shinya Yamanaka and his coworkers tested 24 candidate genes which were known to be pluripotency-associated. After elimination of irrelevant factors, a minimum of four factors remained that were minimally required to generate mouse iPSCs. These factors are Octamer 3/4 (OCT3/4), SRY-box containing gene 2 (SOX2), cytoplasmic Myc protein (c-MYC), and Krueppel-like factor 4 (KLF4) [30]. Specific ESC markers including Oct3/4, Nanog, E-Ras, Cripto, Dax1, and Zfp296 and Fgf4 were used to confirm that pluripotent stem cells were obtained. Only a year later, the successful derivation of hiPSCs from fibroblasts was reported by two different groups. Yamanaka's group used retroviral vectors encoding OCT4 (also known as Pou5F1), SOX2, KLF4, and c-MYC while the group of James Thomson used lentiviral vectors encoding OCT4, SOX2, NANOG, and Lin-28 to reprogram human fibroblasts for revealed of iPS cells [31, 32].

### 2.2. IPSC Technology

**Figure 1:**
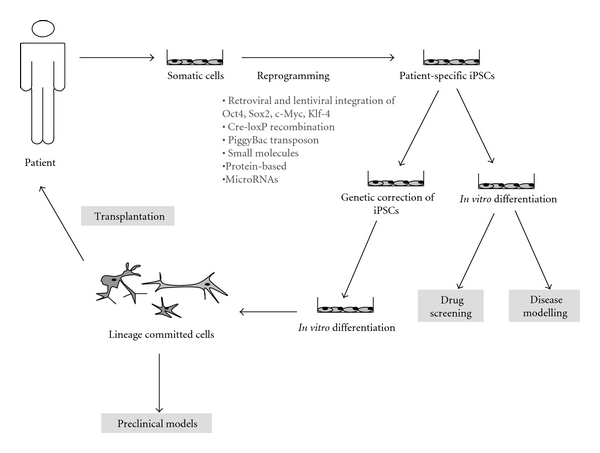
Potential applications of patient-specific pluripotent stem cells in neurological diseases.

Generation of iPCS from somatic cells is a complicated process that is affected by many factors such as the source of the initial cell type (type of the somatic cell used for reprogramming), the particular cocktail of factors used for reprogramming, as well as the methods for delivery of reprogramming factors and culture conditions (see Figure 1). 

#### 2.2.1. Initial Cell Type

 In addition to fibroblasts, iPSCs have now been generated from a large variety of somatic cell populations. Cell types that have been used for iPSCs derivation include keratinocytes [33], pancreatic *β* cells [34], neural cells [35], mature B and T cells [36], melanocytes [37] hepatocytes [38], amniotic cells [39, 40], and cells derived from adipose tissue [41, 42]. However, so far only fibroblasts have been used to generate iPSCs from patients suffering from neurological diseases. 

The inherent differences among the cell types that have been used for reprogramming may affect the efficiency of iPSC generation as well as the quality of the generated iPSCs. For instance, iPSCs derived from mouse embryonic fibroblasts or hepatocytes exhibit a lower tendency to form teratomas when compared to iPSCs derived from mouse tail-tip fibroblasts [43]. Depending on the cell type used for reprogramming, reprogramming can be achieved with different efficiencies and kinetics. For instance, human primary keratinocytes reprogrammed 100-fold more efficiently and twofold faster as compared to human fibroblasts [33]. The kinetics of reprogramming may also vary for different species if related cell types are used. While 20–25 days were necessary to reprogram human skin fibroblasts, only 8–12 days were sufficient for mouse embryonic fibroblasts. Thus, the appropriate choice of cell type is a crucial aspect that should be considered before starting reprogramming experiments: cells used for reprogramming should be accessible easily with minimal risk procedures and should be available in large quantities, in addition to showing high reprogramming efficiencies and iPSC derivation speed.

#### 2.2.2. Reprogramming Factors

Reprogramming factors, such as OCT4, SOX2, KLF4, c-MYC, Nanog, and LIN28, have putative roles in ESC development. OCT4 seems to be the essential reprogramming factor for most initial cell types [44]. Sox2 synergistically activates Oct-Sox enhancers with Oct3/4 and regulates the expression of pluripotent stem cell-specific genes [45]. However, Sox2 in itself is not an essential factor for the generation of iPSCs because the absence of Sox2 can be compensated for by transforming growth factor *β* (TGF *β*) inhibitors or high level of Oct4 [44, 46, 47]. KLF4 is highly expressed in mouse ESCs and constitutes an important factor for reprogramming of mouse somatic cells, yet human fibroblasts could be successfully reprogrammed in the absence of KLF4 [31]. Much of the tumorigenic properties of iPSCs might be caused by c-MYC, which increases the efficiency of iPSC generation but also has ongogenic potential [48]. Instead of c-MYC and KLF, the factors Nanog and LIN28 were also successfully used for reprogramming. Nanog appears not to be an essential factor but it increases the efficiency of reprogramming [49].

As many of the current reprogramming factors tend to endow iPSCs with tumor formation capacity, it is necessary to find new reprogramming factors that lack tumorigenicity. Specific chemicals, such BIX-01294 (BIX), which is a G9a histone methyltransferase (G9a HMTase) inhibitor [50] 5-aza-2′-deoxycytidine (5-azadC), valproic acid (VPA), and kenpaullone [51], have been used to replace oncogenic reprogramming factors [52, 53]. In addition, a wide range of microRNAs, which are crucially involved in maintenance, differentiation, and lineage determination, have recently been identified in ESCs. These studies have shown that microRNAs can contribute to the pluripotency machinery [54, 55] and that they can facilitate reprogramming synergistically with OCT4, SOX2, and KLF4. Most importantly, they can replace c-MYC, which has the greatest tumorigenic potential among reprogramming factors [56]. Recent studies also demonstrated that the p53/p21 pathway plays an important role in iPSC generation and acts as a barrier for tumorigenicity [34, 57–60]. It could be shown that knockdown of p53 results in increased iPSCs generation efficiency. Lin-28, overexpression, which is a negative regulator of miRNA biogenesis, and p53/p21 pathways also increased iPSC formation. Therefore, miRNAs involved in p53 signaling could be used for reprogramming process without genetic modification of the donor cells.

#### 2.2.3. Delivery Method

Retroviral and lentiviral vectors have been widely used for the delivery of reprogramming factors. Critical steps in iPSC generation include that reprogramming factors can be efficiently overexpressed in the somatic cell type that is chosen for reprogramming and that expression of the exogenously applied reprogramming factors is silenced once the cells have been transformed to a pluripotent stage. Retroviruses efficiently infect only proliferating cells, but expression of reprogramming factors is silenced in the ESC stage. Lentiviral vectors on the other hand infect dividing as well as nondividing cells. Yet, a major disadvantage of lentiviral vectors is that reprogramming factors are not efficiently silenced once the cells reach pluripotency. Major disadvantages, which both vector systems have in common, is that they contain oncogenic transcription factors and that they randomly integrate into the genome of infected cells. Adenoviral vectors, which do not integrate into the infected cells genome have also been used to deliver reprogramming factors [34]. Yet, their infection efficiency is much lower than retroviral systems.

 New strategies have been suggested to generate safe and less tumorigenic iPSCs by using nonviral methods or by omitting the oncogenic factors c-MYC and KLF4 [31, 48]. Therefore, attempts have been made to derive iPSCs by using plasmids rather than viruses [61] or by removal of transgenic sequences from the host genome after successful reprogramming using recombination-based excision systems, such as Cre-loxP recombination or PiggyBac transposition [61–63]. Cre-loxP recombination efficiently removes exogenously delivered reprogramming factors from their genomic integration sites in iPSCs [63]. An advantage of the PiggyBac system, however, is that the transposon is able to excise itself without leaving a footprint at the integration locus of the reprogrammed cell genome whereas at least one loxP site remains after Cre-loxP recombination. For this reasons, PiggyBac transposition might be superior to Cre-LoxP recombination. 

Viral free methods have been explored to deliver reprogramming factors by transfecting cells with standard transfection methods, such as liposomes. However, these approaches have a limited efficiency that may be overcome through the recent development of polycistronic vectors. OriP/EBNA1 vectors derived from the Epstein-Barr virus were used to transfect human somatic cells with episomes [64]. However, the efficiency of iPSC generation was very low, and reprogramming factors expression gradually decreased when expressed from OriP/EBNA1 episomal vector transfection. 

Other nonviral delivery methods including RNA and protein transfection have also been tried for iPS generation. Recently, Warren et al. could show that human somatic cells converted to iPSCs by using synthetic mRNAs [65]. This system is simple and efficient, but again has increased oncogenic potential due to relative high levels of c-MYC expression. Protein transduction may be an alternative way for iPSCs generation without genetic interference. Drosophila antennapedia peptide, the herpes simplex virus VP22 protein, and the HIV TAT protein transduction motif are the most widely used proteins in this approach [66]. Besides those, small molecule carriers (SMoCs) [67] and cell-penetrating peptides (CPP) [68, 69] have also been used to carry reprogramming factor proteins into host cells [70].

#### 2.2.4. Culture Conditions

A major problem in iPSC generation for therapeutical us is that xenogenic products are used at multiple steps in current protocols for iPSC generation and maintenance. For instance, fetal bovine serum (FBS) containing media processed with animal-derived enzymes (e.g., trypsin) are used to maintain primary cultures of the human somatic cells that are to be reprogrammed. Xenogenic contamination might also occur when viruses are used to transduce somatic cells with reprogramming factors. Feeder cell layers of mitotically inactivated mouse embryonic fibroblasts plated on gelatin of animal origin and culture media containing serum substitutes, such as knockout serum replacement (KO-SR), are used for the growth and selection of reprogrammed iPSC colonies and their maintenance. Therefore, xeno-free alternatives to those products have been tested for the derivation and maintenance of human embryonic stem cell (hESC) lines [19, 71]. Recently, immortalized human fibroblast lines have been shown to be permissive for iPSC generation [72], and reprogramming of human fibroblasts under xeno-free conditions could be achieved at efficiencies that were similar to conditions when animal-derived products were used [73]. 

For all these reasons, strict quality control procedures related to the culture of iPSCs are crucial, especially if the generated iPSCs are intended to be delivered to human subjects. Another common problem in cell culture work is mycoplasma contamination, and contamination tests should be performed on iPSCs and all other cells that were used during their derivation. This control is particularly critical because mycoplasma infection has been shown to dramatically change stem cell viability and function. Another problem is the tendency of cells in culture to become genetically instable, and karyotype analysis on iPSCs should be performed, particularly after extended serial passaging [23].

#### 2.2.5. Validation of iPSCs

Patient-specific iPSCs should validated and subjected to rigorous quality controls before deemed pluripotent. Therefore, a test for pluripotency should be performed by testing for the presence of pluripotency markers in human iPSCs that will be used in cell theraoy. These markers include cytoplasmic alkaline phosphatase and cell surface markers such as the stage-specific embryonic antigens (SSEA) SSEA-3, SSEA-4 and the tumor recognition antigens (TRA) TRA-1–60 and TRA-1–81. Immunocytochemistry and flow cytometry can be routinely used to assay the expression of these pluripotency markers. Pluripotent iPSCs and hESCs also endogenously express the nuclear transcription factors OCT4, SOX2, and NANOG and endogenous expression of these factors must be distinguished from expression of the exogenously introduced factors that might have been delivered during the reprogramming procedure. Oligonucleotide primers specific for either the endogenous or exogenous factors can be designed and used in RT-PCR in order to solve this problem. In addition, gene expression profiling can also be used to test for the presence of pluripotency markers. Most of the characteristic genes, which are associated with the pluripotent state, have been identified by microarray studies using hESCs. Therefore, the expression of these pluripotency markers can be analyzed by performing multiple standard quantitative RT-PCR, specialized RT-PCR arrays, or by using microarray platforms. Important for cell replacement therapy, the differentiation potential of iPSCs and hESCs should also be tested. A simple method to check for pluripotency of iPSCs and hESCs is to test for their ability to form embryoid bodies (EBs) *in vitro* while a more stringent test would comprise an examination of their teratoma formation potential *in vivo* [23].

#### 2.2.6. Neural Differentiation from iPSCs

The use of iPSCs for the treatment of neurological disorders requires that iPSCs can differentiate into the relevant neuronal subtypes that should be replaced or repaired by the therapy. The extended knowledge of neural development has provided a good opportunity to generate neural cells from iPSCs, and neurons of different parts of the neural tube have been successfully generated, including spinal motoneurons [74], midbrain dopaminergic neurons [75], spinal cord interneurons [76], purkinje and granule cells of the cerebellum [77, 78], hypothalamic neurons [79], and cortical pyramidal neurons [80, 81]. Those studies have revealed that ESC neurogenesis, much like in neural induction during embryonic development, is regulated by the coordinated actions of bone morphogenetic proteins (BMP), Wnt, fibroblast growth factor (FGF), and insulin-like growth factors (IGF) signaling pathways. Neural induction in ESCs and specification of ESC-derived neural progenitors follow the same order of signals as *in vivo*, and proper timing of exposure to these factors can give rise to well-defined neuronal populations. 

Several groups have reported *in vitro* differentiation of neural cells from human iPSCs using the embryoid body formation method. The earliest recognizable cell type in the neural lineage is the neural ectoderm. The differentiation of human iPSCs including patient-specific iPSCs into neural ectoderm cells [82] has been demonstrated by positive staining for NES [83], increased expression of PAX6, and neural cell adhesion molecule mRNAs [31, 84]. Yamanaka's group could also show that human iPSCs can differentiate into *β*III-TUBULIN-positive neurons as well as GFAP-positive astrocytes [32, 48].

#### 2.2.7. The Equivalency of iPSC to ESC

IPSCs and ESCs share major properties such as self-renewal and pluripotency; that is, they are capable of producing cells of all kind of tissues and organs [40, 85]. IPSCs and ESCs have a similar phenotype because their gene expression patterns and epigenetic makeup are highly similar [86, 87]. Recent studies, which compare human and mouse ESCs, however, have shown that being phenotypically similar does not necessarily include that they are functionally equivalent. Because pluripotency is controlled by different signaling pathways in human and mouse ESCs, they cannot be considered to be functionally equivalent [88]. On the contrary, however, it has been demonstrated that human iPSCs and hESCs might rely on the identical signaling pathways [89] in order to ensure their pluripotency and that early cell fate decisions are controlled by similar mechanisms [90]. Therefore, these studies suggest that human iPSCs and ESCs are functionally equivalent.

## 3. IPSCs Derived from the Patients with Neurological Diseases

The generation of human iPSCs offers new approaches to model and cure human diseases. In 2008, Park et al., for the first time, created patient- as well as disease-specific iPSCs from skin fibroblasts of patients that suffered from a variety of genetic diseases, including adenosine deaminase deficiency-related severe combined immunodeficiency, Gaucher disease type III, Duchenne (DMD) and Becker muscular dystrophy (BMD), Parkinson disease (PD), Huntington's the disease (HD), juvenile-onset, type 1 diabetes mellitus, Down syndrome (DS)/trisomy 21, and the carrier state of Lesch-Nyhan syndrome [82]. Similarly, bone marrow mesenchymal cells of a male patient with Shwachman-Bodian-Diamond syndrome were used to create iPSCs. Disease-specific genetic defects have been characterized in iPSCs derived from patients suffering from monogenic diseases. Patient-specific iPSCs share major morphological, molecular, and developmental features with human ESCs, they form teratomas in immunodeficient mice and show multilineage differentiation capacity [82]. The differentiation of patient-specific iPSCs into specific neural cell types, however, has not been investigated in the study by Park et al. (2008). Subsequently, several groups have also successfully generated a wide range of iPSCs from patients with genetic, metabolic, cardiac, and hematological diseases [101, 102]. A list of neurological disorders, including neurodevelopmental and neurodegenerative diseases, that were used to generate patient-specific iPCSc is summarized in Table 1. 

### 3.1. Early-Onset Genetic Neurological Disorders

Certain monogenic neurological diseases are characterized by young childhood-onset, such as spinal muscular atrophy (SMA) and familial dysautonomia (FD). These diseases are autosomal recessive and are caused by the loss of function of a single specific gene, and rapid disease progression occurs within the first years of life. SMA is a group of autosomal recessive diseases caused by loss or mutations in the survival motor neuron (SMN) genes [24]. SMA type 1 is caused by mutations in the survival motor neuron1 gene (SMN1), leading to reduced SMN protein levels and a selective dysfunction of motor neurons, although the pathogenesis of the disease and its specificity to the spinal motor neurons are not fully understood. Recently, Ebert et al. successfully established iPSCs from a type 1 SMA patient and his unaffected mother, and showed that patient-specific iPSCs expanded robustly in culture and retained the capacity to generate differentiated neural tissue and motor neurons while maintaining the disease genotype and phenotype of selective motor neuron death [91]. Although the behavior of these cells *in vivo* remains to be identified, patient-specific iPSCs represent a novel and promising resource to study the mechanisms of SMA. Further functional studies, including electrophysiological experiments and coculture studies with motor neurons derived from SMA-iPSCs and muscle fibers, are needed. It has been proposed that more iPSC clones from other patients and control cases will reduce the concern that the observed phenotype could be a consequence of intrinsic iPSC variability [24, 91]. 

Lee and coworkers recently established iPSCs from another fatal neurodegenerative disease, FD, that results from an aberrant splicing of the IkB kinase complex-associated protein (IKBKAP) [92]. FD, also known as hereditary sensory and autonomic neuropathy III (HSAN-III) or Riley-Day syndrome, is a sensory and autonomic neuropathy that affects the development and survival of sensory, sympathetic, and parasympathetic neurons [103]. Lee et al. showed that iPSC-derived neural crest precursor cells from three FD patients had low levels of IKBKAP expression and exhibit neuronal differentiation and migration defects [92]. In addition, comparative transcriptome analyses revealed significantly reduced expression levels of several transcripts in FD-derived iPSCs when compared to human control iPSCs. Among the candidates validated by quantitative PCR, many genes were involved in peripheral neurogenesis and neuronal differentiation [92].

Ku et al. have reported the derivation of patient-specific iPSCs from skin fibroblasts of two Friedreich's ataxia patients by transcription factor reprogramming [94]. Friedreich's ataxia is an autosomal recessive neurodegenerative disease caused by a mutation in the FXN gene encoding the mitochondrial protein frataxin [104]. The neurodegeneration in the dorsal root ganglia, accompanied by the loss of peripheral sensory nerve fibres and degeneration of the posterior columns of the spinal cord, is a hallmark of the disease and is responsible for the typical combination of signs and symptoms that are specific to Friedreich's ataxia [105]. The mutant FXN gene contains GAA·TTC triplet repeat hyperexpansions within the first intron. Long GAA·TTC repeats cause heterochromatin-mediated gene silencing and loss of frataxin expression in FD patients. Ku et al. showed that FXN gene repression is maintained in FD patient-specific iPSCs derived from fibroblasts [94]. Using these cells, Ku et al. showed that the silencing of the mismatch repair enzyme MSH2 prevents repeat expansion, providing a possible molecular explanation for repeat instability in Friedreich's ataxia. 

Patient-specific iPSCs have also been derived from diseases in which genomic imprinting is affected and which are accompanied by neurological symptoms [97, 98]. Angelman syndrome results from the loss of the maternal copy of the E3 ubiquitin ligase (UBE3A) and goes along with mental retardation, seizures, sleep disturbance, and ataxia. The sister syndrome, Prader-Willi syndrome, is caused by a similar loss of paternally inherited genes and maternal imprinting. This syndrome is a neurological disorder characterized by neonatal hypotonia, failure to thrive, hypogonadism and short stature, mild to moderate mental retardation, and compulsive hyperphagia in early childhood that leads to morbid obesity [98]. Chamberlain et al. derived iPSCs from patients with Angelman and Prader-Willi syndrome, providing a promising cellular model of these neurogenetic disorders [97]. The authors were able to differentiate functional neurons from a total of three iPSC lines. IPSC derived from the patients with Prader-Willi syndrome retain high level of DNA methylation in the imprinting center of the maternal allele and show concomitant reduced expression of the disease-associated small nucleolar RNA HBII-85/SNORD116 [98]. 

Two research groups have recently established iPSCs from patients with Down's syndrome (DS), a frequent genetic cause of mental retardation in humans, occurring in 1 out of 700 births [82, 99]. This syndrome is an autosomal chromosomal abnormality caused by the presence of an additional third copy of chromosome 21 and is also known as trisomy 21. IPSCs derived from skin fibroblasts of two DS patients showed the characteristic trisomy 21 anomaly [82]. Baek et al. injected iPSCs generated from a DS individual into an immunodeficient mouse [99]. The resulting teratomas show trisomy 21, which results in suppression of angiogenesis while control mice had considerable tumor growth due to blood vessel formation. These experiments provide an explanation for the relatively low incidence of most solid tumors in DS patients. Consistently, the expression of DS candidate region-1 (DSCR1), which encodes a protein that suppresses vascular endothelial growth factor-mediated angiogenic signaling by the calcineurin pathway, was increased in DS tissues [99].

### 3.2. Late-Onset Genetic Neurological Disorders

In addition to early-onset genetic disorders, patient-specific iPSCs have also been obtained from patients with late-onset neurodegenerative diseases including HD, PD, and ALS. Interestingly, fibroblasts obtained from elderly patients could be reprogrammed with similar efficiency as those obtained from younger patients [62, 95]. Park et al. showed the presence of expanded (CAG)n polyglutamine triplet repeat sequences in the proximal portion of the huntingtin (Htt) gene in iPSCs derived from a HD patient [82]. HD is a dominantly inherited fatal neurodegenerative disease caused by a CAG repeat expansion in the first exon of the gene Htt. The genotype and phenotype of neurons derived from HD patient-specific iPSCs was recently extensively investigated by Zhang et al. [93]. Currently there is no cure for HD. The HD-specific iPSC line studied by Zhang et al. was originally derived from an HD patient with a 72-repeat CAG tract in the study by Park et al. [82]. HD-specific iPSCs can be differentiated to NSCs and further to striatal neurons exhibiting the genotypic and phenotypic changes that are specific to HD [93]. The endogenous HD mutation persisted in all cell types, and the stability of the CAG trinucleotide repeats in NSCs and striatal-differentiated neurons derived from iPSCs ensures the consistency and reproducibility for the use of these cells as HD model. NSCs derived from HD-specific iPSCs show enhanced caspase 3/7 activity and cellular toxicity upon growth factor withdrawal compared to normal control NSCs [93]. 

In contrast to HD, the vast majority of ALS and PD cases are sporadic. Yet, the study of rare familial forms of these neurodegenerative diseases can also provide valuable information about the pathogenesis of age-related sporadic forms of ALS and PD as both familial and sporadic cases share common phenotypic traits, such as the involvement and loss of specific neuron subpopulations. ALS is a progressive fatal neurodegenerative disease affecting upper and lower motor neurons. In 2008, Dimos et al. generated patient-specific iPSCs derived from skin fibroblasts of two elderly sisters with ALS-associated mutations in the gene encoding superoxide dismutase (SOD1) [95]. One of the cases clinically exhibited the signs of ALS. Using an *in vitro* differentiation protocol, the authors differentiated motor neurons from embryoid bodies formed by patient-specific iPSCs. Within the same cultures glial cells could also be identified. Genotypic analysis revealed a L144F polymorphism in SOD1 gene both in patient-specific iPSCs and iPSC-derived motor neurons [95]. However, disease-specific phenotypic pathologies in the differentiated neurons and glial cells remain to be identified. Following the generation of iPSCs from skin fibroblasts of a sporadic PD patient by Park et al., another recent study showed the generation of iPSCs from the skin biopsy materials of five sporadic PD patients [62, 82]. The authors were successful in differentiating the iPSC lines into human dopaminergic neurons. Using the same iPSC lines, a more recent study has further optimized the differentiation protocol to obtain human ventral midbrain dopaminergic neurons [96].

## 4. Modeling Neurological Diseases with Patient-Specific iPSCs

In neurology, most of the current knowledge about disease-related neuronal phenotypes is gathered from postmortem studies because obtaining live brain tissue is limited. This creates a problem for understanding disease progression and development, because postmortem samples only represent the end-stage of the disease. In addition, aspects of the pathology that are observed in these samples could be secondary and not faithfully reflect the exact disease phenotype on a cellular level [24]. Therefore, knowledge of human neuropathological abnormalities and their progression during the course of a disease is similarly limited. Interspecies differences make it difficult to accurately simulate human neurological diseases in animal models. Therefore, disease modeling by recapitulating the diseases phenotype *in vitro* and in defined cell populations is an important advancement and would make it possible to understand cellular mechanisms of the neurodegenerative diseases [23]. Reprogramming of somatic cells which are taken from rare, monogenic neurogenetic disorders or familial and sporadic multifactorial neurodegenerative disease backgrounds offer a unique opportunity for patient-specific studies and for studies on the cellular level *in vitro* [25, 82]. Naturally, investigation of multifactorial diseases has been more challenging because of their more complex genetic backgrounds and because they are usually influenced by environmental factors [24]. Many studies have been reported that include the generation of iPSCs-derived neural cells but only few of them were able to recapitulate the phenotype of a disease in the iPSC-derived neural population. 

Incomplete disease modeling using iPSCs has first been reported in autosomal recessive neurodegenerative diseases by Ebert et al. it showed that SMA-iPSCs-derived neural cells undergo selective neuronal death, which could be reversed by adding the compounds known to raise the production of SMN protein [91]. However, fully functional neurons showing the disease phenotype could not be produced. Although these cells show a decreased amount of mRNA transcripts, reduced SMN protein levels could not be demonstrated in adult cell type. This study is a proof-of-concept study for iPSCs technology but an incomplete disease model in itself [91]. FD results from an aberrant splicing of IKBKAP transcripts, which has recently been modeled using patient-specific iPSCs [92]. In this study defective IKBKAP splicing decreased neurogenesis and affected migration of iPSCs derived neural precursors. However, similar to the SMA study, decreased protein production could not be observed in fully mature cell [92]. 

Subsequently, iPSCs have also been established from the patients with late-onset neurodegenerative diseases. Dimos et al. successfully directed differentiation of iPSCs which were generated from cells of two familial ALS patients. The iPSCs were able to differentiate into motor neurons expressing appropriate motor neuron markers such as Hb9 and ISLET but no disease phenotype was observed [95]. The generation of patient-specific iPSCs from sporadic ALS cases has not been reported so far. Jaenish and his colleagues reported that dopaminergic neurons could be derived from iPSCs that were generated from sporadic PD patients [62]. However, no gross loss of dopaminergic neurons was reported, indicating that additional stressors may be required to reveal phenotypes of neurodegenerative diseases with late-onset. A vast majority of PD cases are sporadic; however, the study of rare family forms of the disease that are associated with specific gene mutations can provide valuable information on the general disease mechanisms, which might be relevant for sporadic forms as well. Different genes including *α*-synuclein (SNCA), PARKIN, and DJ-1 have been associated with familial PD cases. It would be interesting to see whether iPSCs or their derivatives generated from familial PD cases could exhibit specific disease genotypes and phenotypes *in vitro*. 

## 5. Drug Screening with Patient-Specific iPSCs and Their Derivatives

Another potential impact of human iPSC research is the use of patient-specific cells for the process of drug discovery. 

The ability to analyze drug responses *in vitro* provides an invaluable tool for testing candidate neurotherapeutic agents in patient-specific iPSC-based disease models [106].

The drug discovery process is time consuming and costly because of high attrition rates and protracted research and development cycles. Many candidate drugs that have significant effect on animal models fail to show significant benefits in clinical trials [29]. Tests based on human cells would avoid potential interspecies variations and, as such, predict more precisely any potential adverse effects in humans [24]. High-throughput drug screening using immortalized human cell lines was a major step forward for the development of new drug therapies. Although immortalized are cheap, easy to grow, and reproducible, they may not accurately reflect the normal physiological conditions [29]. Therefore, human iPSCs and their differentiated derivatives hold a great promise for gaining new insights into the mechanisms of human neurological diseases and for drug screening. Patient-specific iPSCs posses a significant advantage as they faithfully reflect the patient's genetic background, the affected cell type, as well as the developmental time [24]. 

The feasibility of exploring the therapeutic action of candidate drugs for human neurological diseases *in vitro* using patient-specific iPSCs has been demostrated. A study by Ebert et al. provides a proof-of-principle for the use of iPSC in drug screening by using the SMA patient-specific iPSCs or their derivatives [91]. In this study iPSC-derived motor neurons were treated with valproic acid or tobramycin, two drugs that increased the expression of both full-length and truncated versions of the SMN protein. SMA-specific iPSCs treated with either of these drugs also showed a significant increase in SMN protein levels compared to untreated control cells. This exciting observation suggests that similar approaches could be used to test the efficacy of candidate drugs before they are administered to patients. Subsequently, Lee et al. (2009) tested the effects of three candidate drugs on aberrant IKBKAP splicing, neuronal differentiation, and migration defects in FD-specific iPSCs and neural crest precursor cells (NCPCs) derived from these iPSCs [92]. The plant hormone kinetin resulted in a marked reduction of the mutant IKBKAP splice form whereas epigallocatechin gallate or tocotrienol exposure did not provide significant improvements. The kinetin-mediated decrease in mutant IKBKAP was associated with an increase in correctly spliced IKBKAP levels and the ratio of normal to mutant transcript [92]. Although short-term (1 or 5 day) kinetin treatment of FD-iPSC-derived NCPCs did not result in a prominent increase in neuronal differentiation or improve neuronal migration, long-term (28 days) kinetin treatment induced a significant increase in the percentage of differentiating neurons. However, kinetin had no effect on NCPC migration suggesting an incomplete restoration of the disease phenotype [92]. These results show the great potential of patient-specific iPSCs in drug screening for the treatment of human neurological diseases. 

Possible toxic effects of candidate drugs are often missed in rodent cells and animal models in general due to specific interactions with human biological processes that cannot be accurately recapitulated in these assay systems [29]. Human iPSCs are thus also an important tool for toxicological testing of drugs and for the identification of environmental factors [107]. The current challenge is the lack of a gold standard for human cell-based toxicity tests. The lessons learnt from the hESC-based assays may be valuable for the standardization and optimization of iPSC-based neurotoxicologic assays. In this context, the choice of reprogramming factors used to reprogram cells, options for reprogramming factor delivery, selection of target cell type, conditions for reprogramming of cells, and culture conditions should be thoughtfully considered for the establishment of iPSCs-based toxicity tests [107]. Beside establishing standards for quality control, technical challenges such as low efficiency, high heterogeneity and variability, and time-consuming and costly methods for the generation and maintenance of iPSCs need to be solved. It also remains to be determined whether artificial *in vitro* systems such as iPSC-based assays can actually faithfully predict the toxicity of drugs in a complex *in vivo* setting [29].

## 6. Therapeutic Potential of Patient-Specific iPSCs in Neurological Diseases

As summarized above, the recent advances in deriving iPSCs from patients offer new and exciting possibilities for biomedical research and clinical applications, as these cells could be used for autologous transplantation in neurological diseases. 

There are two main strategies for using iPSCs to treat neurological disorders. The first is to produce new neurons to replace those lost during disease progression. The second is to produce glial cells that could protect neurons from ongoing degeneration by expressing and secreting critical humoral factors, for example, growth factors. Park et al. showed that growth factor-expressing glial cells can contribute to motor neuron protection in ALS mice. This study underlines the important fact that (micro-) environmental conditions should also be considered in cell-based therapies and might include the delivery of glial cell types along with neurons. 

As summarized before, iPSCs derived from patients with chronic neurological diseases can be successfully differentiated into different types of neural cells, neuronal subtypes, or neural cell precursors *in vitro* [13, 62, 91–93, 95, 115]. In addition, the results of *in vivo* experimental transplantation studies suggest that engrafted neurospheres derived from human iPSCs are able to differentiate to various types of neural cells such as neurons, astrocytes, and oligodendrocytes in the central nervous system of embryonic mice [114]. Upon transplantation into the fetal mouse brain, NPCs derived from mouse iPSCs migrate into various brain regions and differentiate into glia and neurons, including glutamatergic, GABAergic, and catecholaminergic subtypes [110]. IPSCs or their derivatives are thus capable to integrate into preexisting functional neuronal circuitries in the CNS. Electrophysiological recordings and morphological analysis demonstrate that the grafted neurons display normal neuronal activity and are functionally integrated into the host brain. These results support the idea that the transplantation of iPSCs or iPSC-derived NSCs could be efficiently used to treat chronic neurological diseases or for neurorestoration in the chronic phase of acute neurological diseases such as stroke and neurotrauma. Indeed, several recent studies that tested this idea in experimental models of stroke, PD, and SCI indicate the therapeutic potential of human or rodent iPSCs (Table 2). The results of a few studies also suggest that transplanted iPSCs or their derivatives are capable of participating and integrating with the preexisting functional neuronal circuitries in the CNS. 

Fetal tissue transplantation for PD has given way to cell replacement therapy for neurodegenerative diseases [116]. PD is mainly caused by the degeneration of mesencephalic substantia nigra dopaminergic neurons and a progressive loss of dopaminergic neurotransmission in the caudate and putamen of the neostriatum [117]. Patients exhibit motor dysfunction such as tremor, rigidity, and bradykinesia, as well as disturbances in sleep and cognition. To date, L-DOPA and other dopamine agonists provide relief of major symptoms but are only effective in the early course of the disease. Deep brain stimulation of the subthalamic nuclei is an additional therapeutic option for PD patients, but requires surgical intervention. While all of these treatments provide symptomatic relief, none of them is able to change the course of the disease or inhibit its progression [5, 117]. Thus, there is a clear need for restorative and regenerative approaches, including cell-based therapies. The restoration of dopaminergic neurons in patients with PD via implantation of embryonic midbrain tissue was taken from animal experiments to clinical applications, but showed only a limited efficacy. Recent studies from several groups have reported the beneficial effect of iPSCs transplantation on behavioral deficits in 6-hydroxydopamine- (6-OHDA) lesioned rats as evaluated by amphetamine- and apomorphine-induced rotational asymmetry [108–110]. Human iPSC line, PD-specific iPSCs, or mouse iPSCs were used in these studies. Transplanted human iPSC-derived midbrain dopaminergic cells survived long term in the graft region and gave rise to midbrain dopaminergic neurons [108]. The PD-specific iPSC-derived dopaminergic neurons survived at high numbers, showed arborization, and mediated functional effects in 6-OHDA-lesioned rats [109]. However, only a few DA neurons developed projections into the host striatum at 16 wk after transplantation whereas PD-specific iPSC-derived nondopaminergic neurons sent axonal projections to specific near and remote target areas in the adult brain. Wernig et al. pursued a different strategy and transplanted mouse iPSC-derived dopaminergic neurons [110]. The contamination of undifferentiated iPSCs and subsequent teratoma formation after transplantation was a major complication that was revealed in this study. They concluded that the contamination of undifferentiated cells was the most likely reason viral transcripts were not detected in teratoma tissues. The risk of tumor formation from the grafted cells was minimized by separating contaminating undifferentiated pluripotent cells from committed neural cells using fluorescence-activated cell sorting [110]. Following the transplantation of human iPSC-derived dopaminergic neurons to 6-hydroxydopamine-lesioned rats, no teratoma formation was observed [19]. These results suggest the importance of the developmental stage or level of differentiation of transplanted cells for the success of cell-replacement studies. 

 Despite advances in medical and surgical care, current clinical therapies for SCI are limited. SCI can affect relatively young people who often become a huge burden to themselves and society due to high medical costs throughout their lives. Rationales for therapeutic use of stem cells for SCI include replacement of damaged neurons and glial cells, secretion of trophic factors, regulation of gliosis and scar formation, and enhancement of axon regeneration [14]. However, the availability of clinically suitable cell sources for human application has been hindered by both technical and ethical issues [15]. The potential therapeutic effect of the cell-replacement by iPSCs in SCI has recently been shown by Tsuji et al. in mouse spinal contusive injury model [114]. When the authors sorted the safe (without contamination of undifferentiated iPSCs) iPSC-derived neurospheres and transplanted them into the mouse spinal cord 9 days after contusive injury, these cells gave rise to all three neural lineages without forming teratomas or other tumors.

 Furthermore, transplanted cells also contributed to endogenous remyelination, induced the axonal regeneration and axonal outgrowth of host serotonergic fibers, and promoted the recovery of locomotor function. In contrast to this, the transplantation of iPSC-derived neurospheres sorted as unsafe exhibited teratoma formation and even had detrimental effects on the functional recovery following SCI [114]. The therapeutic potential of iPSCs in traumatic brain injury remains unexplored.

Acute stroke is the second or third most common cause of death and the primary cause of morbidity and long-term neurological disability worldwide. The only FDA-approved treatment for stroke is the use of the thrombolytic agent, tissue-plasminogen activator. This therapy is very limited due to a narrow therapeutic time window and the risk of hemorrhagic complications. The regeneration of the brain after damage is active even weeks after stroke occurs, which might provide a rational for cell-replacement therapy [116]. Transplantation of stem cells or stem cell-derived progenitors has long been seen as a therapeutic solution to repair the damaged brain and shown to be safe and effective in ischemic stroke animal models. However, conclusive evidence in patients is still lacking. As only small randomized controlled trials with ischemic stroke patients exist. Thus, it is too early to know whether stem cell transplantation can improve the functional outcome and comprehensive, well-designed trials are needed [10]. The derivation of human iPSCs may offer a new possibility for cell-based therapies of stroke-induced brain damage. In a rat stroke model (middle cerebral artery occlusion), Chen et al. showed that the direct injection of iPSCs into damaged areas of rat cortex significantly decreased the infarct size and improved the motor function as evaluated by the animals performance in rotarod and grasping tasks [111]. Furthermore, subdural transplantation of iPSCs mixed with fibrin glue as a less invasive delivery route could also effectively reduce the total infarct volume and greatly improve the behavior of cerebral ischemic rats. This treatment regime also had positive effects on attenuating inflammatory cytokine expression due to cerebral ischemia. However, contrary results were obtained in another study using a similar cerebral ischemia model in mice, which was not able to show any beneficial effect of mouse iPSC transplantation [112].

 Moreover, transplanted undifferentiated iPSCs formed tridermal tumorigenesis in ischemic mouse brain, suggesting a facilitating and promoting effect of the postischemic microenvironment for the development of tumors. IPSCs grafted into ischemic brains formed teratoma with higher probability and larger volume as compared to those formed in intact brain tissue. The expression of matrix metalloproteinase-9 and phosphorylated vascular endothelial growth factor receptor2 are significantly increased in iPSC-derived tumors in the ischemic mouse brain [113]. The effect of postischemic microenvironment on tumorigenesis may be specific to the differentiation status of transplanted cells similar to those shown for hESC-derived NPCs [118]. These results suggest that the safety of iPSCs should be critically evaluated not only under normal condition, but also in specific pathological conditions such as experimental cerebral ischemia. Daadi et al. showed the potential of human ESC-derived NSCs in a neonatal brain hypoxia animal model [119]. Hypoxic-ischemic brain injury in newborn infants is a major cause for cerebral palsy, neurological disability, and epilepsy. Stem cell-based therapy has the potential to rescue and replace the ischemic tissue caused by hypoxic-ischemic injury and to restore function of the nervous system [120]. The relevance of these findings for the use of iPSCs remains to be studied. The generation of iPSCs from human neonatal tissues may offer a promising cell source for cell-replacement therapy in infants [121]. 

## 7. Limitations of iPSC Technology

### 7.1. Limitations of Generating of iPSC

As outlined above, the major limitation of iPSC-based therapies is its tumorigenic potential. Current differentiation methods are not efficient enough to transform all iPSCs, and undifferentiated cells remain in the preparation [110, 122, 123]. This problem can be overcame by designing positive-negative selection using fluorescent-activated cell sorting (FACS) or drug selection approaches [114]. 

Most patient-specific iPSCs have been generated by lentiviruses or retroviruses containing reprogramming factors, which both lead to genomic integration of the transgene and which may not get silenced efficiently at the pluripotent stage. Moreover, reactivation of the silenced transgenes can cause undesirable side effects in reprogrammed cells. Thus, avoiding genomic integration may be a crucial step in the derivation of safe iPSCs for cell replacement therapy and can be achieved by improving transgene-free or nonviral methods [124, 125]. 

Several groups put considerable effort in working out nonviral delivery methods, such as using chemicals that can improve efficiency in order to derive iPSCs [52, 70, 126, 127]. One of these studies showed that a chemical approach could significantly improve (200-fold) the efficiency of viral iPSC generation from human fibroblasts therefore providing a way towards the development of safer, more efficient, and nonviral methods for reprogramming human somatic cells [128].

For regenerative medicine, human iPSCs and hESCs are promising cell sources. They have the common ability to differentiate into all three germ layers; however, one recent study revealed that heterogeneity in gene expression levels is much greater among hiPSCs than among hESCs. It is also suggested that human iPSCs occupy an alternate and less stable pluripotent state. Moreover, human iPSCs display slower growth kinetics and impaired directed differentiation when compared to hESCs [129].

According to recent studies, many mouse iPSCs are also prone to epigenetic abnormalities and sustain a transient epigenetic memory of their donor cells [130, 131]. Therefore, together with development of optimized differentiation methods, a careful analysis of genomic and epigenetic integrity of human iPSCs is required for generating homogenous adult-like cells capable of *in vivo* function. In addition, the variability that has long been observed between different human ESC lines can be even more dramatic in human iPSCs because of their diverse origins and the different modes of derivation [132]. Thus, variability between human iPSCs requires the development of specific protocols for nearly every iPSC line generated. In two recent studies, neurons were generated from fibroblasts by directed reprogramming without an intermediary ESC-like state [133, 134]. In addition to cell reprogramming without reversion to a pluripotent stem cell state, *in vivo* reprogramming has also been reported [135]. Thus, a better understanding of the mechanisms controlling reprogramming of somatic cells such as transdifferentiation bears the potential for faster and wider practical applications in cell therapy than iPSCs might. 

Another challenge of iPSC technology is that the process of well-characterized and carefully validated iPSC line generation is a time-consuming process. The lag between the initiation of reprogramming and cell delivery to a patient might render the process inappropriate for therapeutic applications, for instance in acute organ failure or injury [124].

In addition to the difficulty of isolating the optimal cell type that has the best potential for transplantation, poor survival rates, limited self-renewal, and limited homing/migration after transplantation should be taken into consideration to improve iPSCs based-approaches [136, 137].

### 7.2. Clinical Limitations of iPSCs in Neurological Disorders

IPSCs cell-based therapies are still in their infancy. Therefore, numerous hurdles must be overcome before they can be used in clinical applications. 

So far, iPSCs technology has been used to investigate monogenetic neurodegenerative diseases and some promising data have been published providing a new way to understand the pathology of the diseases. However, multifactorial neurodegenerative disease modeling has not been performed yet. One of the questions concerning iPSC-based disease modelling is whether late-onset diseases like AD and PD can be efficiently recapitulated *in vitro* within a few days of cell culture or whether the exposure to different types of environmental factors or genetic stress to the cells is necessary for revealing the desired disease phenotype. One study related to PD showed that iPSCs derived from sporadic PD patients do not display apparent abnormalities when compared with wildtype neurons [62]. Most of the neurodegenerative diseases develop in a noncell-autonomous manner requiring the interaction of different cell types [4, 138]. Recent findings indicate that astrocytes may play a role in the specific degeneration of spinal motor neurons in ALS [139, 140]. The effect of different cell types on disease development were studied in mouse and human ALS models. ESCs were engineered to carry ALS-specific SOD1 mutation and differentiated into both motor neurons and astrocytes that were supposed to interact during disease development. The coculture of both cells types caused a prominent cell death in motor neurons indicating that astrocytes contribute to the pathophysiology of ALS [141–143]. Noncell autonomous mechanisms have also been implicated in other neurodegenerative disorders such as spinocerebellar ataxia [144, 145]. Thus, efficient coculture systems should be developed to test for these effects. However, current technologies for differentiation of iPSCs are limited to a few functional cell types and are not very efficient. In neurodegenerative disease modeling from iPSCs, genetic information, environment, and senescence all take part in the neurodegeneration process and thus achieving relevant conditions *in vitro* would be crucial.

## 8. Future Prospects

Despite a large body of current research, iPSC technology is still in its infancy. There are many limitations that have to be solved before clinical trials for treatment of neurological disorders can be performed. For monogenic diseases, which require gene targeting to repair mutant alleles, new targeting strategies need to be developed concomitantly [100]. A recent study reported that the use of zinc-finger nucleases can correct genetic defect by homologous recombination with high efficiency in iPSCs and hESCs [146] and may be an appropriate and efficient method for correction of genetic defect in human cells.

 IPSCs technology needs to be standardized in order to create medically relevant cells. For the translation of iPSCs into therapeutics and human clinical applications, current good manufacturing practices (cGMP) will also be necessary [106]. The scientific community, the government, and/or private organizations are responsible for the implementation of guidelines. This is essential to accelerate the global movement towards safer iPSCs in an academic, clinical, or private sector.

 So far, most of the iPSCs lines have been exposed to animal products either in direct or indirect ways, which could make these cells improper for transplantation.

 Therefore, cGMP standards have to construct standardized animal-free methods for the derivation of iPSC lines [106]. Human iPSCs can be generated under serum and feeder cell-free conditions bringing iPSCs an important step closer to clinical applications [21, 147]. 

An alternative to the feeder cell-free culture method has been established by using autologous skin fibroblasts derived from the same patient, which provides an appropriate source of feeder cells [148, 149]. Additionally, as well as developing serum/feeder-free conditions and autogenic feeder cells, effective cryopreservation procedures are essential to facilitate cell banking for future applications [150].

Nonviral and transgene-free reprogramming methods have been significantly improved, yet the cancer-causing potential of safe iPSCs still needs to be evaluated in animal models before clinical applications in humans. Besides small animals such as mice and rats, iPSC therapies need to be validated in large animal models which are anatomically and physiologically more relevant to humans. IPSCs have been generated from monkeys [151–153] and pigs [154, 155] which are better models for preclinical transplantation studies.

The safety and efficacy of cell-based therapies for neurodegenerative diseases also depend on the method of cell administration. One study has suggested that intranasally administered cells could pass through the blood-brain barrier [156, 157]. Therefore, this kind of noninvasive methods for cell delivery to the CNS needs to be further explored for iPSCs.

## 9. Conclusion

Since the first generation of human iPSCs, a large number of patient-specific iPSC lines have been developed. Recently, iPSC studies have exploded and the potential application of these cells in establishing disease models, drug screening, and cell transplantation-based therapy have been widely recognized. IPSCs have distinct advantages over hESC such as the lack of ethical restrictions and immune rejection of the graft. In addition to their direct therapeutic use in cell replacement therapy, disease modeling is most likely the most important aspect of iPSC technology. IPSCs are the patient's own cells and therefore the best possible source for cell transplantation treatment provided that their genetic program can be changed for the treatment of neurodegenerative diseases. Human iPSCs hold great promise for the treatment of incurable neurodevelopmental and neurodegenerative diseases. Furthermore, iPSC-based cell therapy may contribute to neurorepair in the chronic phase of acute CNS injuries such as stroke and neurotrauma. However, iPSC technology still faces specific difficulties such as low efficiency and high variability. Finally, iPSC-based therapeutic strategies need to be evaluated in preclinical animal models of neurological diseases before clinical trials.

##  Conflict of Interests

The authors declare no competing financial interests.

## Figures and Tables

**Table 1:** Patient-specific pluripotent stem cells in neurological diseases.

Disease	Disease gene/molecular defect	Generated neural cell type	Disease-specific genotype/phenotype in iPSCs and/or generated neural cells	Reference
SMA (type 1)	SMN	Motor neuron	Decreased neuronal survival	Ebert et al. [91]
FD	IKBKAP	NCPC	Impaired neuronal differentiation and migration	Lee et al. [92]
HD	Huntingtin	Striatal neuron	Enhanced caspase activity and neurotoxicity	Zhang et al. [93]
		NSC	Upon growth factor deprivation in iPSC-derived NSCs 72 CAG repeats in iPSCs	Park et al. [82]
FA	Frataxin	—	GAA·TTC triplet repeat instability in iPSCs	Ku et al. [94]
ALS	Multifactorial	Motor neuron	L144F polymorphism of SOD1 gene	Dimos et al. [95]
PD	Multifactorial	vmDopaminergic neuron	Not evaluated	Cooper et al. [96]
		Dopaminergic neuron	Not evaluated	Soldner et al. [62]
			Not evaluated	Park et al. [82]
AS	UBE3A	Neuron/astrocyte	UBE3A repression	Chamberlain et al. [97]
PWS	Imprinting defect	Neuron/astrocyte-like	Decreased SNORD116 expression in iPSCs	Yang et al. [98]
		—	Methylation imprint in iPSCs	Chamberlain et al. [97]
DS	Trisomy 21	—	Decreased tumor formation by iPSCs	Baek et al. [99]
			Trisomy 21 in iPSCs	Park et al. [82]
BMD	Dystrophin	—	Not shown	Park et al. [82]
DMD	Dystrophin	—	Deletion of exons 45–52 in iPSCs	Park et al. [82]
		—	Deletion of exons 4–43 in iPSCs	Kazuki et al. [100]

Amyotrophic lateral sclerosis (ALS), Angelman syndrome (AS), Becker muscular dystrophy (BMD), Down syndrome (DS), Duchenne muscular dystrophy (DMD), familial dysautonomia (FD), Friedreich's ataxia (FA), Huntington disease (HD), I-*κ*-B kinase complex-associated protein (IKBKAP), neural crest precursor cell (NCPC), neural stem cell (NSC), Parkinson disease (PD), Prader-Willi syndrome (PWS), small nucleolar RNA (snoRNA) HBII-85 (SNORD116), survival motor neuron (SMN), superoxide dismutase 1 (SOD1), spinal muscular atrophy (SMA), ubiquitin protein ligase E3A (UBE3A), and ventral midbrain (vm).

**Table 2:** iPSC-based cell-replacement therapy in preclinical animal models of neurological diseases.

Disease	Species	Model	Transplanted cells	Delivery route	Outcome	Reference
PD	Rat	6-OHDA	mbDA PGCs derived from hiPSCs	Transplantation	Long-term survival Differentiation to DA neurons Tumor-like cells at the site of graft	Cai et al. [108]
	Rat	6-OHDA	PD patient iPSC- derived DA neurons	Transplantation	Improved motor behavior	Hargus et al. [109]
	Rat	6-OHDA	iPSC-derived DA neurons	Transplantation	Improved motor behavior	Wernig et al. [110]
	Rat	6-OHDA	iPSC-derived DA neurons	Transplantation	Improved motor behavior	Swistowski et al. [19]
Stroke	Rat	MCAO	iPSCs + FG	Direct injection to infarct area/subdural	Decreased infarct size Improved motor performance Decreased inflammatory cytokines	Chen et al. [111]
	Mouse	MCAO	Mouse iPSCs	Transplantation	Tridermal tumorigenesis	Kawai et al. [112]
	Mouse	MCAO	Mouse iPSCs	Transplantation	Increased teratoma risk and volume Increased MMP9 and pVEGFR2	Yamashita et al. [113]
SCI	Mouse	Contusion model	iPSC-derived neurospheres	Transplantation (contusion area)	Remyelination and functional recovery	Tsuji et al. [114]

6-hydroxydopamine (6-OHDA), dopaminergic (DA), fibrin glue (FG), induced-pluripotent stem cell (iPSC), matrix metalloproteinase-9 (MMP9), midbrain (mb), middle cerebral artery occlusion (MCAO), Parkinson's disease (PD), phosphorylated vascular endothelial growth factor receptor2 (pVEGFR2), progenitor cell (PGC), and spinal cord injury (SCI).
